# Supplementary material for: Nanostructure of Porous Si and Anodic SiO2 Surface Passivation for Improved Efficiency Porous Si Solar Cells
Source: Nanomaterials (Basel). 2021 Feb 11;11(2):459. doi: 10.3390/nano11020459 (PMC7916900; doi:10.3390/nano11020459)
Supplement: Supplementary file 1 [file nanomaterials-11-00459-s001.pdf]

nanomaterials

Supplementary Materials

Nanostructure of Porous Si and Anodic SiO_2 Surface Passivation for Improved Efficiency Porous Si Solar Cells

Panus Sundarapura [1], Xiao-Mei Zhang [1,2,*], Ryoji Yogai [3], Kazuki Murakami [3], Alain Fave [4] and Manabu Ihara [1,3,*]

1. Department of Chemical Science and Engineering, Tokyo Institute of Technology, 2-12-1 Ookayama, Meguro, Tokyo 152-8552, Japan; sundarapura.p.aa@m.titech.ac.jp
2. Department of Mechanical Engineering, Tokyo Institute of Technology, 2-12-1 Ookayama, Meguro, Tokyo 152-8552, Japan
3. Department of Chemistry, Tokyo Institute of Technology, 2-12-1 Ookayama, Meguro, Tokyo 152-8552, Japan; energy.power.ry.516@gmail.com (R.Y.); kazuki.techtech@gmail.com (K.M.)
4. Univ. Lyon, INSA Lyon, Institut des Nanotechnologies de Lyon (INL), UMR 5270, INSA de Lyon, 69621 Villeurbanne CEDEX, France; alain.fave@insa-lyon.fr

* Correspondence: xiaomeizhang2007@gmail.com (X.-M.Z.); mihara@chemeng.titech.ac.jp (M.I.)

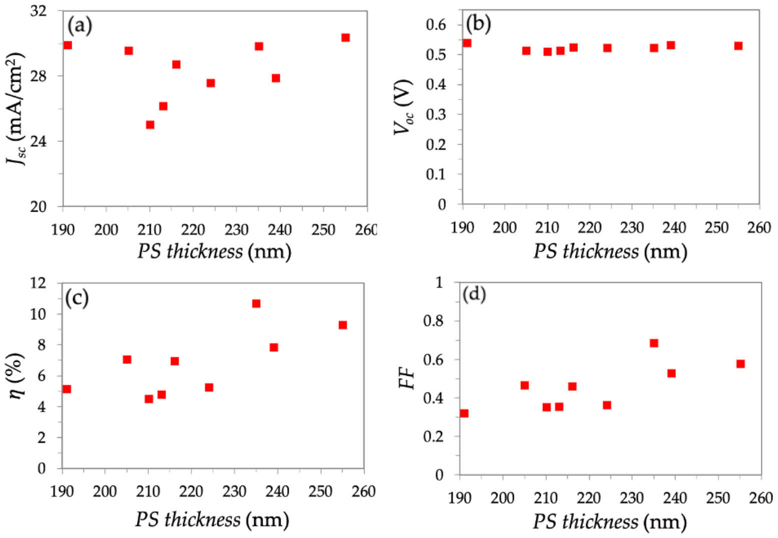

Figure S1. Dependence of PS thickness on PV parameters of PS-2 cells: (**a**) J_{sc}; (**b**) V_{oc}; (**c**) η; (**d**) *FF*; (**e**) *FF*.

© 2021 by the authors. Licensee MDPI, Basel, Switzerland. This article is an open access article distributed under the terms and conditions of the Creative Commons Attribution (CC BY) license (http://creativecommons.org/licenses/by/4.0/).
